# Recent Advances in Nanoenzymes Based Therapies for Glioblastoma: Overcoming Barriers and Enhancing Targeted Treatment

**DOI:** 10.1002/advs.202413367

**Published:** 2025-01-24

**Authors:** Liyin Wang, Min Gu, Xiaoli Zhang, Tingting Kong, Jun Liao, Dan Zhang, Jingwu Li

**Affiliations:** ^1^ Shengjing Hospital of China Medical University Liaoning 110004 China; ^2^ Shenyang Sport University Liaoning 110102 China; ^3^ Institute of Systems Biomedicine Beijing Key Laboratory of Tumor Systems Biology School of Basic Medical Sciences Peking University Beijing 100191 China; ^4^ The First Hospital of China Medical University Liaoning 110001 China

**Keywords:** blood–brain barrier, glioblastoma, nanoenzymes, nanomedicine

## Abstract

Glioblastoma multiforme (GBM) is a highly aggressive and malignant brain tumor originating from glial cells, characterized by high recurrence rates and poor patient prognosis. The heterogeneity and complex biology of GBM, coupled with the protective nature of the blood–brain barrier (BBB), significantly limit the efficacy of traditional therapies. The rapid development of nanoenzyme technology presents a promising therapeutic paradigm for the rational and targeted treatment of GBM. In this review, the underlying mechanisms of GBM pathogenesis are comprehensively discussed, emphasizing the impact of the BBB on treatment strategies. Recent advances in nanoenzyme‐based approaches for GBM therapy are explored, highlighting how these nanoenzymes enhance various treatment modalities through their multifunctional capabilities and potential for precise drug delivery. Finally, the challenges and therapeutic prospects of translating nanoenzymes from laboratory research to clinical application, including issues of stability, targeting efficiency, safety, and regulatory hurdles are critically analyzed. By providing a thorough understanding of both the opportunities and obstacles associated with nanoenzyme‐based therapies, future research directions are aimed to be informed and contribute to the development of more effective treatments for GBM.

## Graphical abstract

This paper summarizes the clinicopathological mechanisms of gliomas and provides a comprehensive overview of the effective treatment of gliomas by nanoenzymes based on strategies such as immunotherapy, enhanced chemotherapy, photothermal therapy, and combination therapy, either through effective drug delivery or enhancement of their own properties. In addition, the translational limitations and challenges of nanoenzymes for the treatment of gliomas are comprehensively discussed in this paper.

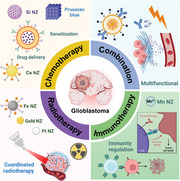

## 1 Introduction

GBM remains one of the most aggressive and intractable forms of cancer, characterized by a median survival time of ≈14 months and a 5‐year survival rate of less than 5%.^[^ ^1^, ^2^, ^3^ ^]^ Despite significant advances in medical science, these grim statistics have remained largely unchanged over the past five decades, underscoring the urgent need for more effective therapeutic strategies.^[^ ^4^, ^5^, ^6^ ^]^ The current clinical standard of care which includes maximal surgical resection followed by adjuvant radiotherapy and chemotherapy with temozolomide remains inadequate for several reasons.^[^ ^7^, ^8^, ^9^ ^]^ First, surgical intervention often fails to completely remove the infiltrative glioma cells deeply embedded within the brain parenchyma, leading to high recurrence rates in GBM patients. The diffuse nature of GBM allows tumor cells to migrate along white matter tracts and blood vessels, making total resection virtually impossible without causing significant neurological deficits.^[^ ^10^, ^11^, ^12^ ^]^ Additionally, the tumor microenvironment (TME) of GBM is highly complex and contributes to therapeutic resistance.^[^ ^13^ ^]^ Hypoxia is a hallmark of GBM due to rapid tumor growth outpacing the development of adequate vasculature, leading to regions of low oxygen tension. Hypoxic conditions promote the stabilization of hypoxia‐inducible factors (HIFs), which activate genes involved in angiogenesis, metabolism, and invasion, further enhancing tumor aggressiveness.^[^ ^14^ ^]^ Moreover, GBM exhibits elevated levels of reactive oxygen species (ROS), which can induce DNA damage and genomic instability, contributing to tumor progression and resistance to therapy. Second, the BBB acts as a formidable obstacle, preventing most chemotherapeutic agents from reaching therapeutic concentrations within the central nervous system (CNS).^[^ ^15^, ^16^, ^17^, ^18^ ^]^ The BBB's selective permeability is essential for maintaining CNS homeostasis but poses a significant challenge for drug delivery to brain tumors.^[^ ^19^, ^20^, ^21^, ^22^ ^]^ Furthermore, GBM cells are known to develop resistance to chemotherapy and radiotherapy through various mechanisms, including enhanced DNA repair capacity, activation of survival signaling pathways, and expression of drug efflux transporters.^[^ ^23^ ^]^ These factors collectively reduce the efficacy of conventional treatments and contribute to poor patient outcomes. Moreover, the intricate and delicate architecture of the brain makes it exceedingly challenging to eradicate cancerous cells without inflicting collateral damage on healthy neural tissue, potentially resulting in severe cognitive and functional impairments. Consequently, there is an urgent imperative to develop novel therapeutic approaches that are both highly effective and safe to improve outcomes for GBM patients.

Nanomedicine has garnered considerable attention in recent years for its potential to enhance targeted cancer therapies while minimizing adverse side effects.^[^ ^24^, ^25^, ^26^, ^27^, ^28^ ^]^ Nanoparticle‐based approaches have demonstrated promise across various cancer types by improving drug solubility, enhancing permeability and retention effects, and enabling controlled drug release.^[^ ^29^, ^30^, ^31^, ^32^ ^]^ However, despite these advances, there remains a paucity of highly specific and efficient nano diagnostics and therapeutics tailored to address the unique challenges posed by GBM. Nanoenzymes—a class of nanomaterials that mimic the catalytic functions of natural enzymes have emerged as promising tools in medical imaging, disease diagnostics, and cancer treatment.^[^ ^33^, ^34^, ^35^, ^36^ ^]^ Their catalytic activities can be engineered to modulate biochemical pathways within the TME, offering innovative mechanisms for cancer therapy.^[^ ^37^, ^38^, ^39^, ^40^, ^41^ ^]^ Due to their nanoscale size, nanoenzymes exhibit the enhanced permeability and retention (EPR) effect, allowing them to preferentially accumulate at tumor sites.^[^ ^42^, ^43^, ^44^, ^45^ ^]^ This selective accumulation helps to overcome the limitations imposed by the BBB, as nanoenzymes can be engineered to cross the BBB via receptor‐mediated transcytosis or other transport mechanisms.^[^ ^46^ ^]^ When combined with advanced drug delivery systems, nanoenzymes can amplify the clinical potential of therapeutic agents by improving their stability, bioavailability, and targeted distribution.^[^ ^47^, ^48^, ^49^ ^]^ Unlike traditional chemotherapeutic agents, nanocarriers can prolong drug retention within tumor tissues, significantly mitigating systemic toxicity and enhancing treatment efficacy.^[^ ^50^, ^51^, ^52^ ^]^ Several nanomedicine formulations have already received clinical approval for various cancers, highlighting the translational potential of this approach.^[^ ^53^, ^54^, ^55^, ^56^ ^]^ Furthermore, nanoenzymes offer a versatile platform for surface modification and functionalization, facilitating the development of advanced drug‐delivery technologies.^[^ ^57^, ^58^, ^59^ ^]^ Their unique physical and chemical properties including tunable optical, electrical, and magnetic characteristics make them ideal candidates for a range of innovative therapeutic modalities.^[^ ^60^, ^61^, ^62^ ^]^ These modalities encompass diagnostic imaging, radiotherapy, photothermal therapy (PTT), photodynamic therapy (PDT), and chemodynamical therapy (CDT), each exploiting different aspects of nanoenzyme functionality to target cancer cells.^[^ ^63^, ^64^, ^65^, ^66^, ^67^, ^68^ ^]^ By incorporating nanoenzymes into nanodrug delivery systems, it is possible not only to improve drug delivery but also to modulate the TME, which is crucial for enhancing the efficacy of immunotherapy. Recent developments in targeted nano diagnostic and therapeutic agents have demonstrated substantial promise in various cancer treatments, including gliomas, by enabling precise tumor targeting and minimizing off‐target effects.^[^ ^69^, ^70^, ^71^ ^]^

**Figure 1:**
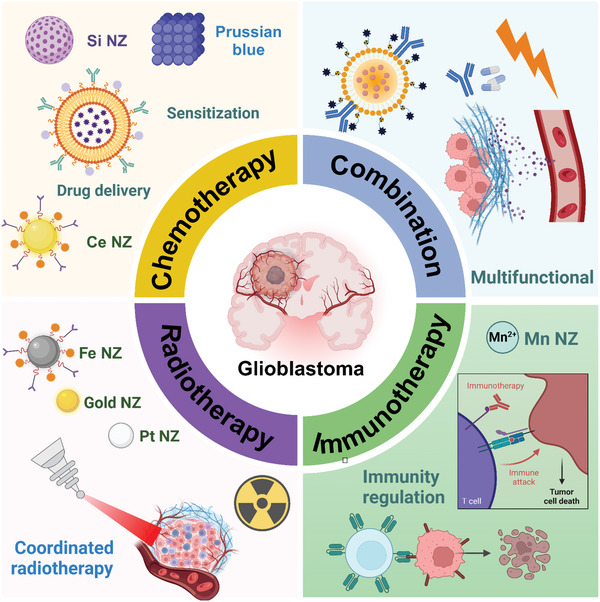
Schematic illustration of effective treatment of GBM through drug delivery, immunotherapy, enhanced chemotherapy, photothermal therapy, etc., based on different nanoenzymes.

In this review, we undertake a comprehensive analysis of the latest advancements in glioma treatment, focusing on both conventional therapeutic approaches and innovative nanomedicine‐based strategies. By critically comparing these modalities, we aim to elucidate their relative strengths, limitations, and the challenges involved, particularly concerning clinical translation. A key focus of this review is the BBB, which continues to represent a significant hurdle in the effective treatment of GBM. We explore the potential of various nanoenzyme‐based therapies including PTT, radiotherapy, immunotherapy, and multidimensional combination therapies and examine how these nanoenzyme delivery systems can efficiently traverse the BBB, ensuring precise and safe diagnosis and treatment of gliomas. We also discuss strategies for enhancing BBB permeability, such as receptor‐mediated transcytosis and temporary disruption techniques, to facilitate nanoparticle delivery. Finally, we provide an in‐depth discussion on the complexities of transitioning nanoenzyme‐based therapies from the experimental stage to clinical application, identifying key challenges such as scalability, biocompatibility, and regulatory considerations, and highlighting critical areas for further research. Through this review, we aim to offer fresh perspectives on the transformative potential of nanomedicine in advancing the therapeutic landscape for GBM (**Figure** **1**).

## 2 Mechanistic Features of GBM

**Figure 2:**
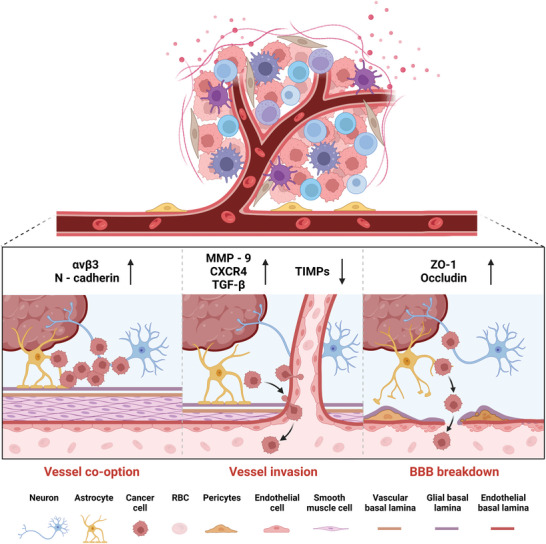
Schematic illustration of the changes in the vasculature and BBB after glioma genesis.

GBM is classified as a grade IV glioma by the World Health Organization, the most aggressive form of brain cancer. It accounts for ≈50% of all gliomas and has an annual incidence of 3 to 8 per 100 000 people.^[^ ^72^ ^]^ While the causes of GBM remain largely unknown, risk factors such as exposure to ionizing radiation are associated with its development. Unlike pediatric gliomas, which often originate in the brainstem, adult gliomas typically develop in the frontal and temporal lobes. GBM shows a slight male predominance, and its incidence increases with age. Although surgical resection is the cornerstone of treatment, it is rarely curative due to the tumor's infiltrative nature, which makes complete removal impossible. Postoperative therapies, including radiotherapy and chemotherapy, are essential to prolong survival, but even with aggressive treatment, the prognosis remains poor. The median survival of GBM patients ranges from 8 to 15 months, with a 5‐year survival rate of only 5%.^[^ ^73^ ^]^ It is the leading cause of death from primary brain tumors due to its extreme malignancy, poor prognosis, and propensity for recurrence.^[^ ^74^, ^75^, ^76^ ^]^ Drug resistance in GBM arises from a complex set of biological and mechanistic features. As shown in **Figure** **2**, after glioma genesis, tumor progression is closely linked and interlocked with a series of vascular‐related processes that together shape its malignant phenotype and influence drug resistance. During tumor initiation, GBM cells primarily utilize vascular co‐selection mechanisms. During this process, tumor cells establish a strong connection with the host vasculature through the adhesion of GBM cells to endothelial cells mediated by adhesion molecules such as integrins. For example, integrin αvβ3 recognizes and binds to ligands such as fibronectin on the surface of vascular endothelial cells, causing tumor cells to attach to the vessel wall.^[^ ^77^ ^]^ At the same time, with the help of a vascular endothelial growth factor (VEGF)‐independent approach, tumor cells were able to use host blood vessels to obtain oxygen and nutrients, achieving rapid expansion along the existing blood vessels and forming a local tumor cell population. During this period, as the angiogenic process is not initiated, the immune surveillance that may be triggered by neovascularization is avoided to a certain extent, allowing tumor cells to proliferate in a relatively “safe” environment. As the tumor size increases, its vascular phenotype changes to become more aggressive. Tumor cells begin to secrete matrix metalloproteinases (MMP), particularly MMP‐2 and MMP‐9, which act as zinc‐dependent endopeptidases that specifically degrade extracellular matrix components such as collagen IV (MMP‐2 action) and laminin (MMP‐9 action) of the vascular basement membrane, resulting in structural disruption and weakening of the vascular wall.^[^ ^78^ ^]^ At the same time, GBM cells undergo adaptive morphological and cytoskeletal changes within the cell, extending pseudopods and enhancing interactions with endothelial cells. With hemodynamic assistance, tumor cells were able to invade the vascular lumen, constructing a channel for subsequent intravascular dissemination and distant metastasis. During tumor progression, GBM cells also induce disruption of the BBB. On the one hand, VEGF and other factors secreted by tumor cells can down‐regulate the tight junction proteins between endothelial cells, such as Claudin‐5 and Occludin, so that the expression of these proteins can be reduced, thus destroying the structure of endothelial cell tight junction.^[^ ^79^ ^]^ At the same time, ZO‐1 as a scaffolding protein of the tight junctions may be displaced and dysfunctional due to tumor‐related factors, resulting in the down‐regulation of its normal tight junction function and impairment of the integrity of the BBB. On the other hand, inflammatory factors (e.g., interleukin‐1β, tumor necrosis factor‐α) and vasoactive substances (e.g., VEGF itself) released by tumor cells increase vascular permeability, and BBB disruption not only encourage more tumor cells to infiltrate into the blood circulation and enhance metastatic potential, but also promotes the infiltration of peripheral immune cells into the tumor microenvironment, as well as the influx of nutrients, which further contributes to the growth, invasion, and immune evasion of tumors., invasion and immune evasion behavior. In summary, the three interrelated processes of vascular co‐selection, vascular invasion, and BBB destruction form a vicious circle in the development of GBM. Tumor cells flexibly switch vascular utilization strategies at different stages, from early co‐selection to late invasion and BBB destruction, and each step lays the foundation for the aggravation of malignancy and the emergence of drug resistance, which makes the treatment of GBM difficult, and also provides a key mechanism and direction of thought for in‐depth research and conquest of GBM drug resistance.

1. Hypoxia. GBM is one of the most vascularized human tumors; however, despite its dense vasculature, it suffers from inefficient microcirculation. This paradox creates extensive hypoxic regions within the tumor. Tumor hypoxia is a critical feature that drives GBM's aggressive behavior.^[^ ^80^ ^]^ Hypoxia promotes resistance to chemotherapy and radiotherapy and induces genetic changes that support tumor survival and proliferation.^[^ ^81^, ^82^, ^83^ ^]^ It also exacerbates genomic instability, increasing mutation rates and enabling the tumor to evolve rapidly. The lack of sufficient oxygen supply in hypoxic areas upregulates HIFs, which stimulate the expression of angiogenic factors like VEGF. This process, known as the “angiogenic switch,” is essential for tumor growth but leads to poorly organized and leaky blood vessels, further impairing drug delivery and therapeutic efficacy.^[^ ^84^ ^]^ Moreover, hypoxia is closely linked to the promotion of invasive behavior and stemness within the tumor, creating a more treatment‐resistant and metastatic phenotype.
2. High Invasiveness. GBM is notorious for its highly invasive nature, which is a key reason for its poor prognosis and high recurrence rates.^[^ ^85^, ^86^ ^]^ Unlike metastatic brain tumors that originate elsewhere in the body, GBM invades surrounding healthy brain tissue aggressively. This infiltrative behavior often leads to a “butterfly” pattern of tumor spread across both hemispheres, complicating surgical removal. Glioma cells do not respect anatomical boundaries, infiltrating deep into the brain parenchyma, including critical regions such as the subventricular zone, which houses neural stem cells. Glioma stem cells (GSCs), a subset of cells within the tumor, are believed to be responsible for driving this invasion.^[^ ^87^, ^88^, ^89^ ^]^ GSCs exhibit enhanced motility and express high levels of MMPs, particularly membrane type 2 matrix metalloproteinase, which degrades the extracellular matrix (ECM) to facilitate invasion. Additionally, GSCs secrete factors that remodel the ECM and generate actin‐rich protrusions known as invadopodium, further enabling the tumor to infiltrate surrounding tissues.^[^ ^90^ ^]^ This highly invasive phenotype renders complete surgical resection nearly impossible, leading to inevitable recurrence even after aggressive surgery.^[^ ^91^ ^]^
3. Drug Resistance. GBM's resistance to conventional treatments, including chemotherapy and radiotherapy, is a major challenge.^[^ ^92^, ^93^, ^94^ ^]^ The standard chemotherapeutic agent for GBM is TMZ, an alkylating agent that induces DNA damage by adding methyl groups to guanine residues. However, resistance to TMZ often develops due to the expression of DNA repair enzyme O6‐methylguanine‐DNA methyltransferase (MGMT), which removes the methyl groups, thereby reversing TMZ's cytotoxic effects. Furthermore, the hypermutated phenotype seen in some gliomas, driven by mismatch repair deficiencies, leads to enhanced resistance to chemotherapy and radiation. In addition to these intrinsic resistance mechanisms, GBM cells employ efflux pumps, such as the ATP‐binding cassette transporters, to actively expel chemotherapeutic agents, thereby reducing their intracellular concentration and effectiveness.^[^ ^95^, ^96^ ^]^ GBM's highly heterogeneous nature further complicates treatment, as different cell populations within the tumor may respond differently to therapies. Tumor hypoxia also contributes to therapeutic resistance by reducing the efficacy of radiation therapy (RT), which relies on oxygen to generate DNA‐damaging free radicals.
4. Immunosuppression. GBM‐induced immunosuppression significantly undermines the efficacy of immunotherapeutic strategies.^[^ ^97^, ^98^, ^99^ ^]^ The GBM TME is characterized by the presence of various immunosuppressive cells, including tumor‐associated macrophages (TAMs), myeloid‐derived suppressor cells (MDSCs), and regulatory T cells (Tregs).^[^ ^100^, ^101^, ^102^ ^]^ These cells suppress antitumor immune responses, allowing the tumor to evade immune surveillance. TAMs, which are abundant in GBM, are typically skewed toward the M2 phenotype, which promotes tumor growth by secreting anti‐inflammatory cytokines, promoting angiogenesis, and facilitating tissue remodeling. Similarly, MDSCs inhibit the activation of effector T cells by secreting immunosuppressive factors such as transforming growth factor‐beta and ROS, while Tregs directly suppress the activity of cytotoxic T cells (CD8+) and helper T cells (CD4+), further dampening the immune response. Hypoxia within the tumor also exacerbates immunosuppression by enhancing the recruitment and activation of these immunosuppressive cells through pathways such as STAT3 signaling. This immunosuppressive environment is a significant obstacle to the success of immunotherapies such as checkpoint inhibitors in GBM.
5. Heterogeneity and Genetic Complexity. One of the most challenging aspects of GBM treatment is its extreme heterogeneity.^[^ ^103^, ^104^, ^105^ ^]^ GBM tumors are composed of a diverse population of cells with varying genetic, epigenetic, and phenotypic profiles. This heterogeneity exists not only between different patients (intertumoral heterogeneity) but also within a single tumor (intertumoral heterogeneity). As a result, no single therapeutic approach can effectively target all tumor cells. Some subpopulations may be sensitive to certain treatments, while others may remain resistant, leading to treatment failure and tumor recurrence. The molecular heterogeneity of GBM is reflected in the diverse range of mutations and alterations found in key signaling pathways, including the receptor tyrosine kinase (RTK)/RAS/PI3K pathway, the p53 pathway, and the retinoblastoma pathway. These pathways regulate critical cellular processes such as proliferation, apoptosis, and cell cycle progression, and their dysregulation drives GBM's growth and resistance to therapy.
6. BBB and BBTB. A key factor is the complex TME of GBM, which plays a crucial role in tumor progression, invasion, and resistance to therapy. The TME consists of various cellular components, including tumor cells, endothelial cells, pericytes, astrocytes, microglia, immune cells, and ECM components. Interactions within the TME promote angiogenesis, suppress immune responses, and enhance tumor cell survival and invasion. Moreover, the hypoxic conditions often found in the GBM TME contribute to the upregulation of genes associated with angiogenesis and treatment resistance. Addressing the TME is therefore essential for developing effective GBM therapies. The BBB and the blood–brain tumor barrier (BBTB) represent major obstacles to the effective delivery of therapeutic agents to the brain.^[^ ^106^, ^107^, ^108^ ^]^ The BBB is a highly selective barrier that tightly regulates the passage of substances between the bloodstream and the brain, protecting the CNS from toxins but also limiting the entry of many therapeutic drugs.^[^ ^109^ ^]^ In the context of GBM, the BBTB adds an additional layer of complexity. While the tumor vasculature is often leaky, allowing some permeability, the BBTB still restricts the entry of large molecules and many chemotherapeutic agents, reducing their efficacy. Additionally, the heterogeneous nature of the BBTB means that drug penetration varies across different regions of the tumor, with some areas being more protected than others. The TME in GBM further complicates treatment by interacting with the BBB and BBTB. The TME can induce changes in endothelial cells and pericytes, altering tight junction integrity and influencing BBB permeability. Hypoxia within the TME leads to the secretion of VEGF and other cytokines that promote angiogenesis and vascular remodeling, affecting BBB function.^[^ ^110^ ^]^ Moreover, the TME contributes to an immunosuppressive milieu that hinders the effectiveness of immune‐mediated therapies. Nanoenzymes offer promising solutions in this scenario by targeting both the BBB/BBTB and the TME. Due to their enzyme‐mimicking catalytic activities, nanoenzymes can modulate the TME to enhance therapeutic efficacy. For instance, nanoenzymes with ROS‐scavenging capabilities can alleviate oxidative stress within the TME, reducing inflammation and mitigating damage to normal brain tissue.^[^ ^111^ ^]^ Conversely, nanoenzymes designed to generate ROS selectively within tumor cells can induce apoptosis, overcoming resistance mechanisms. Furthermore, nanoenzymes can be engineered to cross the BBB more effectively. Surface modification with targeting ligands such as transferrin, lactoferrin, or angiopep‐2 allows nanoenzymes to exploit receptor‐mediated transcytosis pathways, enhancing their penetration into the brain. Once past the BBB, nanoenzymes can accumulate within the TME and exert their therapeutic functions, such as disrupting the ECM to improve drug penetration or reprogramming immune cells to mount an antitumor response. By addressing both the physical barriers of the BBB/BBTB and the complex interactions within the TME, nanoenzymes hold the potential to significantly improve drug delivery and efficacy in GBM treatment.^[^ ^112^ ^]^ Their multifunctional capabilities enable them to overcome obstacles that have traditionally limited the success of GBM therapies. Overcoming the BBB, BBTB, and the challenges posed by the TME is a key focus in GBM therapy. Novel drug delivery systems, including nanoparticle‐based therapies and nanoenzymes, are being developed to enhance the delivery of therapeutics across these barriers. The integration of nanoenzymes into treatment strategies represents a promising advancement, as they can simultaneously modulate the TME, improve BBB penetration, and deliver therapeutic agents effectively to tumor sites.

## 3 Current Treatments and Limitations of GBM

The current standard of care for GBM, which includes maximal safe surgical resection followed by radiotherapy and concurrent chemotherapy with TMZ, has remained largely unchanged for ≈2 decades. Although these approaches provide temporary control of tumor progression, they have had limited success in extending long‐term survival due to GBM's aggressive and highly invasive nature. Moreover, the presence of the BBB and BBTB hinders the delivery of therapeutic agents, further complicating effective treatment. In this section, we discuss the key therapeutic modalities, their mechanisms, and the significant limitations that continue to challenge GBM treatment.

### 3.1 Surgical Resection

Surgical resection is the first‐line therapy for GBM and aims to achieve maximal tumor debulking while preserving essential neurological functions.^[^ ^113^, ^114^, ^115^ ^]^ Several studies have demonstrated that the extent of resection is directly correlated with patient survival; the more tumor tissue removed, the better the prognosis.^[^ ^116^, ^117^, ^118^ ^]^ However, achieving complete resection is nearly impossible due to the diffuse infiltration of tumor cells into surrounding healthy brain tissue. These infiltrating cells remain post‐surgery and are responsible for rapid tumor recurrence, contributing to the median survival rate of less than 15 months. Furthermore, many GBM tumors invade critical areas of the brain responsible for functions such as language, motor control, and sensory processing, making aggressive resection potentially harmful to the patient.^[^ ^119^ ^]^ To enhance the precision of surgical resection, intraoperative imaging techniques have become essential. Preoperative magnetic resonance imaging (MRI) is commonly used to visualize tumor boundaries, but it has limitations in detecting microscopic tumor infiltration. Intraoperative MRI (iMRI) provides real‐time imaging during surgery, allowing surgeons to adjust their resection strategy as the operation progresses. This technology can account for the anatomical changes caused by the surgery itself, thereby improving tumor removal accuracy. Another advancement is fluorescence‐guided surgery (FGS) using 5‐aminolevulinic acid (5‐ALA). 5‐ALA is metabolized by tumor cells into protoporphyrin IX, which fluoresces under ultraviolet light, enabling the surgeon to differentiate between tumor tissue and normal brain tissue. FGS has significantly improved the ability to achieve more extensive resections and has been associated with better progression‐free survival outcomes. However, even with advanced imaging technologies and surgical precision, complete resection remains unattainable for most GBM patients due to the highly infiltrative nature of the tumor.^[^ ^120^ ^]^

### 3.2 Radiation Therapy

RT is a critical component of GBM treatment and is typically administered after surgery to target residual tumor cells.^[^ ^121^, ^122^, ^123^ ^]^ The standard course of RT involves a total dose of 60 Gy delivered over six weeks, fractionated into daily doses of 2 Gy. Radiation works by inducing DNA damage in rapidly dividing cells, leading to cell death. However, GBM's inherent radio resistance significantly limits the effectiveness of RT.^[^ ^124^ ^]^ Tumor hypoxia, which is common in GBM, further diminishes the sensitivity of glioma cells to radiation by reducing the production of ROS, which is necessary for radiation‐induced DNA damage. In recent years, advancements in RT techniques have aimed to improve precision and minimize damage to surrounding healthy brain tissue.^[^ ^125^ ^]^ 3D conformal radiation therapy (3D‐CRT) and intensity‐modulated radiation therapy (IMRT) allow for more precise targeting of the tumor by shaping the radiation beams to conform to the tumor's geometry.^[^ ^126^ ^]^ IMRT, in particular, modulates the intensity of radiation, delivering higher doses to the tumor while sparing critical structures such as the optic nerves and brainstem. Stereotactic radiosurgery and stereotactic radiotherapy are also increasingly used in GBM management, particularly for smaller tumors or recurrent diseases. These techniques deliver high‐dose radiation in a single or a few sessions, achieving greater precision and reducing overall treatment time. Nevertheless, despite these technological improvements, the highly heterogeneous nature of GBM, coupled with its radio resistance, limits the long‐term effectiveness of RT. The treatment often controls tumor growth temporarily, but recurrence is nearly inevitable due to residual radioresistant tumor cells.

### 3.3 Chemotherapy

TMZ remains the most widely used chemotherapeutic agent in the treatment of GBM, primarily because of its ability to cross the BBB.^[^ ^127^, ^128^, ^129^, ^130^ ^]^ TMZ is an oral alkylating agent that induces DNA damage by adding methyl groups to the O6 position of guanine residues. This DNA damage triggers apoptosis in tumor cells, but the efficacy of TMZ is often limited by the presence of the DNA repair enzyme O6‐methylguanine‐DNA methyltransferase (MGMT). Tumors that express high levels of MGMT can repair TMZ‐induced DNA damage, rendering the treatment ineffective. Studies have shown that patients with methylation of the MGMT promoter, which silences MGMT expression, have a better response to TMZ and improved overall survival. The “Stupp Protocol,” which established the current standard of care, involves concurrent TMZ and radiotherapy, followed by adjuvant TMZ. While this regimen has improved median survival from 12.1 to 14.6 months, the overall impact remains modest, and the 5‐year survival rate is still below 10%. In addition, ≈50% of GBM patients do not benefit from TMZ due to MGMT‐mediated resistance.^[^ ^131^ ^]^ For recurrent GBM, TMZ is even less effective, and no standard second‐line therapies have demonstrated significant survival benefits. Researchers have explored combining TMZ with other therapies, including targeted agents and immunotherapies, but these combinations have thus far failed to produce significant clinical improvements. The development of TMZ resistance, the heterogeneity of GBM, and the presence of cancer stem cells that are inherently resistant to chemotherapy contribute to the limited success of current chemotherapeutic approaches.

### 3.4 Blood–Brain Barrier: A Major Obstacle to GBM Therapy

One of the greatest challenges in GBM treatment is the BBB, a highly selective membrane that restricts the passage of most therapeutic agents into the brain.^[^ ^132^ ^]^ The BBB consists of endothelial cells connected by tight junctions, pericytes, and astrocytic end feet, which together create a physical and metabolic barrier that protects the brain from toxins and pathogens.^[^ ^133^, ^134^, ^135^ ^]^ However, this also prevents most chemotherapeutic agents, biologics, and large molecules from reaching therapeutic concentrations within the brain. The BBTB develops as GBM grows and disrupts the integrity of the BBB.^[^ ^136^ ^]^ While the BBTB is more permeable than the normal BBB, this increased permeability is inconsistent across different regions of the tumor. High‐grade gliomas such as GBM exhibit a more disrupted BBTB, but even in these cases, the penetration of therapeutic agents remains limited. For most drugs, only ≈0.1% of the administered dose reaches the brain, necessitating high systemic doses that increase the risk of systemic toxicity.

Beyond the BBB and BBTB, cellular barriers within the TME further impede effective drug delivery. The dense ECM of GBM tumors poses a physical barrier to the penetration of therapeutic agents. The ECM is composed of various proteins, glycoproteins, and proteoglycans that can hinder the diffusion of drugs, especially larger molecules such as nanoparticles and biologics. Moreover, cellular uptake mechanisms can limit the intracellular accumulation of therapeutics.^[^ ^137^ ^]^ Endocytosis, particularly endosomal entrapment, and subsequent lysosomal degradation can reduce the bioavailability of drugs within tumor cells. Nanoparticles and other delivery systems may be internalized by endocytosis but can become sequestered within endosomal compartments, preventing them from reaching their intracellular targets such as the nucleus or mitochondria. Additionally, efflux transporters like P‐glycoprotein (P‐gp) and multidrug resistance‐associated proteins are overexpressed in GBM cells and can actively pump out therapeutic agents, leading to decreased intracellular drug concentrations and contributing to chemoresistance. Endocytose phagocytosis by tumor‐associated macrophages and microglia can also impact drug delivery. These immune cells can internalize nanoparticles and other therapeutic agents, sequestering them away from tumor cells and reducing their efficacy. The heterogeneity of GBM cells, including the presence of cancer stem cells, adds another layer of complexity, as different cell populations may exhibit variable uptake and sensitivity to therapies.^[^ ^138^ ^]^ Several strategies are being investigated to enhance drug delivery across the BBB and BBTB. Nanotechnology‐based approaches, such as nanoparticle carriers and liposomal formulations, aim to improve the transport of therapeutic agents through receptor‐mediated transcytosis or by transiently disrupting the BBB using focused ultrasound.^[^ ^139^, ^140^ ^]^ These methods show promise in preclinical studies but have yet to demonstrate significant improvements in clinical outcomes. Additionally, researchers are exploring the use of convection‐enhanced delivery (CED), a technique that bypasses the BBB by delivering drugs directly into the brain via a catheter.^[^ ^141^, ^142^, ^143^, ^144^ ^]^ CED has been used to deliver chemotherapeutics, biologics, and nanoparticles directly to the tumor site, achieving higher local drug concentrations. However, this approach is invasive and carries the risk of complications such as infection and brain injury. To address cellular barriers, strategies such as co‐administering efflux transporter inhibitors or designing nanoparticles that evade efflux mechanisms are being explored. Modifying nanoparticles to promote endosomal escape, such as incorporating fusogenic peptides or pH‐buffering “proton sponge” materials, can enhance cytosolic delivery of therapeutics. Targeting the TME to degrade the ECM using enzymes like collagenase or hyaluronidase may improve drug penetration into the tumor mass.

While advancements in surgical techniques, radiation therapy, and chemotherapy have improved the management of GBM, the overall prognosis remains poor. The key challenges in GBM treatment include the inability to achieve complete surgical resection, the development of drug and radiation resistance, and the difficulty of delivering therapeutics across the BBB and BBTB. Furthermore, the highly heterogeneous nature of GBM, both within individual tumors and between patients, complicates the development of effective, universally applicable treatments. Future research is focused on overcoming these challenges by developing novel therapeutic strategies. These include personalized medicine approaches that tailor treatments based on the molecular and genetic characteristics of the tumor, immunotherapy to harness the body's immune system to fight GBM, and nanomedicine‐based therapies that improve drug delivery and target specific tumor cells. Addressing both physiological and cellular barriers is essential for the successful translation of these therapies from the laboratory to the clinic. Despite promising advances in these areas, significant hurdles remain, and continued efforts are necessary to improve outcomes for patients with GBM.

### 3.5 Nanomedicines and Cross‐Barrier Strategies

Traditional nanomedicines can encapsulate chemotherapeutic drugs and targeted therapeutic agents inside nanocarriers in various ways, such as liposomal nanoparticles, which can effectively load hydrophilic and hydrophobic drugs, realize the protection of the drugs, and achieve the appropriate drug release curve.^[^ ^145^ ^]^ This can improve the stability of the drugs to prevent them from being degraded before reaching the lesion and carry sufficient amounts of drugs across the BBB to act on the glioma cells. For example, some polymer nanoparticles can encapsulate a commonly used glioma chemotherapeutic drug, such as temozolomide, so that it can be stabilized in the blood circulation and reach the tumor site in the brain.^[^ ^146^ ^]^ The surface modification of the nanomedicine, such as the attachment of targeting ligands (e.g., transferrin and other substances that can bind to relevant receptors on the BBB), can be used to achieve a certain degree of active targeting of the BBB and increase the efficiency of the drug in crossing the BBB to enter the glioma tissues in the brain. Nanoparticles, like those modified to interact with specific transport proteins on the BBB, lend themselves to the transport mechanisms mediated by these proteins for smoother access to the tumor regions in the brain. The materials used to make traditional nanomedicines are rich and diverse, including lipids, polymers, and inorganic materials.^[^ ^147^ ^]^ For example, liposomes are biocompatible, not easily trigger immune reactions in vivo, and the preparation process is relatively mature and simple; polymer nanoparticles can be flexibly adjusted by adjusting the composition and molecular weight of the polymer to regulate their properties, such as the drug release rate and circulation time in vivo; inorganic nanomaterials (e.g., gold nanoparticles) have unique physical properties such as optics and magnetism, which is convenient for subsequent imaging and monitoring applications, and can better observe the distribution of the drug in vivo across the BBB as well as at the glioma, and help to assess the therapeutic effect. In addition to traditional nanomedicines, micro/nanomotor‐driven strategies have emerged as a promising frontier for enhancing drug delivery across the BBB. Micro/nanomotors, including biohybrid motors, can be engineered to move autonomously in response to environmental cues, such as magnetic fields, pH gradients, or chemical gradients (chemotaxis).^[^ ^148^ ^]^ These motors, which often combine biological components (such as bacteria or enzymes) with synthetic nanomaterials, can be designed to actively propel themselves across biological barriers, including the BBB. A notable example is biohybrid motors, which integrate natural biological components with synthetic materials to harness the motility of microorganisms (e.g., bacteria) or biological molecules (e.g., flagella) for targeted delivery.^[^ ^149^ ^]^ Biohybrid motors have shown promise in crossing the BBB, as they can be engineered to respond to specific environmental stimuli present in the brain, such as low pH or hypoxia, which are common features of the GBM microenvironment.

Nanozymes, on the other hand, possess unique advantages over conventional nanoparticle‐based drugs due to their intrinsic catalytic activities similar to those of natural enzymes, such as oxidoreductase and hydrolase activities.^[^ ^150^ ^]^ After crossing the BBB and entering the glioma tissue, nanozymes can not only directly kill tumor cells through catalytic reactions such as generating highly oxidative ROS to induce apoptosis but also regulate the TME. For example, in the microenvironment of glioma, the catalytic reactions of nanozymes can change the local acidity and redox status, affecting the proliferation, invasion, and angiogenesis of tumor cells, and playing a multifaceted therapeutic role. This is an advantage that traditional nanomedicines lack when they rely solely on the loaded drug to exert medicinal effects.^[^ ^151^ ^]^ The multifunctionality of nanozymes allows them to serve both therapeutic and diagnostic purposes within a single nanoplatform. Their sustained catalytic activity under relatively mild physiological conditions provides long‐lasting intervention on glioma cells and the TME, inhibiting tumor development from multiple aspects. Unlike some traditional drugs that may have a short duration of efficacy and require frequent administration, nanozymes offer continuous therapeutic effects without the need for repeated dosing. Nanozymes can also be engineered to enhance targeting ability. By modifying their surfaces with specific ligands or antibodies that recognize overexpressed receptors on glioma cells or BBB endothelial cells, nanozymes can achieve receptor‐mediated endocytosis, facilitating their transport across the BBB and accumulation in tumor tissues. For instance, the transferrin receptor, which is highly expressed in both BBB endothelial cells and glioma cells, can be targeted by nanozymes conjugated with transferrin to improve delivery efficiency.

Protein corona formation is another mechanism by which nanozymes enhance drug delivery across the BBB. When nanozymes enter the bloodstream, proteins adsorb onto their surfaces, forming a “protein corona” that can influence their biological interactions.^[^ ^152^, ^153^, ^154^ ^]^ By controlling the composition of the protein corona through surface modifications, nanozymes can be designed to evade immune recognition and exploit endogenous transport pathways, improving their ability to cross the BBB and target glioma cells. Compared to other nanoparticle‐based drugs like quantum dots, which are primarily used for imaging due to their fluorescent properties, nanozymes offer both therapeutic and diagnostic functions through their catalytic activities. Quantum dots may pose concerns regarding heavy metal toxicity and long‐term safety, whereas nanozymes can be constructed from biocompatible materials that minimize toxicity and immune reactions. Furthermore, nanozymes prepared from natural biomaterials or materials with good biocompatibility such as those based on ferritin or Prussian blue do not easily cause strong immune rejection in vivo. They have better compatibility with the organism and can minimize damage to normal brain tissues, avoiding side effects due to immune reactions when they cross the BBB and enter the brain to play a therapeutic role. This presents certain advantages in terms of safety compared with some traditional nanomedicines that may lead to immune reactions due to the material itself or the drug carried. After being metabolized in the body, the products of nanozymes can be excreted or further utilized by the body relatively easily, unlike some traditional nanomedicines that may remain and accumulate in the body, potentially causing long‐term adverse effects. This reflects their strengths in terms of biosafety.

## 4 Nanoenzymes for GBM Treatment

GBM is one of the most aggressive and lethal forms of brain cancer, with limited effective treatment options and a poor prognosis. Conventional therapies face significant challenges, including poor penetration of the BBB, nonspecific targeting, drug resistance, and severe side effects. In this context, nanoenzymes have emerged as a promising and innovative approach for GBM treatment due to their unique properties and capabilities. Nanoenzymes, artificial enzymes based on nanomaterials, have demonstrated immense potential in the treatment of GBM.^[^ ^155^, ^156^, ^157^, ^158^, ^159^ ^]^ These nanoscale materials mimic the catalytic activities of natural enzymes, offering several key advantages. First, they exhibit higher catalytic stability and are less susceptible to environmental conditions compared to natural enzymes. Natural enzymes can be unstable and degrade under harsh conditions such as extreme pH, high temperatures, or the presence of proteases. In contrast, nanoenzymes maintain robust catalytic functions even in the challenging TME of GBM, making them ideal candidates for cancer therapy.^[^ ^159^, ^160^, ^161^, ^162^ ^]^ Nanozymes can mimic the activities of critical antioxidant enzymes like SOD and CAT, which play vital roles in regulating oxidative stress within cells. In the GBM microenvironment, elevated levels of ROS contribute to tumor progression, angiogenesis, and resistance to therapy. By simulating SOD activity, nanozymes catalyze the dismutation of O₂⁻· into H₂O₂ and molecular O₂, reducing superoxide‐induced damage. Subsequently, CAT‐mimicking nanozymes decompose the generated H₂O₂ into water and O₂, further alleviating oxidative stress and hypoxia within the tumor. For example, cerium oxide (CeO₂) nanoparticles exhibit both SOD‐ and CAT‐mimetic activities due to the presence of mixed valence states (Ce^3^⁺/Ce⁴⁺) on their surface. In the GBM microenvironment, CeO₂ nanozymes can scavenge excessive ROS, protecting normal neuronal cells from oxidative damage while inducing apoptosis in tumor cells through modulation of redox balance.^[^ ^163^ ^]^ Second, nanoenzymes can be engineered to possess multifunctional properties, including therapeutic, diagnostic, and targeting capabilities within a single nanoplatform. This multifunctionality enables them to overcome multiple barriers associated with GBM treatment. For example, nanoenzymes can be designed to cross the BBB efficiently, target tumor cells selectively, and generate ROS to induce apoptosis or necrosis in cancer cells while sparing healthy neuronal tissue. Iron oxide nanoparticles (Fe₃O₄), which possess peroxidase‐like activity, can catalyze the Fenton reaction within the acidic TME of GBM. They convert endogenous H₂O₂ into highly reactive ·OH, leading to oxidative damage and apoptosis of tumor cells a strategy known as CDT. When combined with targeting ligands that recognize GBM‐specific receptors (e.g., transferrin receptors), these nanozymes can selectively accumulate in tumor tissues, enhancing therapeutic efficacy while minimizing off‐target effects.

In addition to their application in GBM, nanozymes have shown promise in other cancer types, including breast cancer and lung cancer.^[^ ^164^ ^]^ In a recent study, CeO₂ nanoparticles were used for the treatment of breast cancer, where they demonstrated significant antitumor effects by modulating oxidative stress and enhancing the efficacy of chemotherapy.^[^ ^165^ ^]^ The CeO₂ nanozymes exhibited peroxidase‐like activity, converting H₂O₂ to highly reactive hydroxyl radicals, which induced selective oxidative stress in tumor cells, resulting in enhanced apoptosis and inhibition of tumor growth. This approach also improved the tumor response to chemotherapy, as the generated ROS sensitized the cancer cells to the chemotherapeutic drug doxorubicin. Similarly, in lung cancer, Fe₃O₄ nanozymes have been applied in chemodynamical therapy, where their peroxidase‐like activity catalyzed the conversion of H₂O₂ into hydroxyl radicals, inducing selective tumor cell death. In a mouse model of lung cancer, Fe₃O₄ nanozymes, when combined with a targeting ligand, successfully targeted the tumor site and significantly reduced tumor volume.^[^ ^166^ ^]^ This study demonstrated the potential of nanozymes in enhancing the therapeutic response by amplifying oxidative stress within the tumor microenvironment. Thirdly, nanoenzymes offer reduced synthesis costs and easier scalability compared to the production of natural enzymes or antibodies, facilitating their potential clinical translation. Their synthetic nature allows for precise control over their composition, size, shape, and surface characteristics, which can be tailored to optimize their performance for specific therapeutic applications. Furthermore, nanoenzymes have already found applications across various biomedical fields, such as tumor therapy, tissue regeneration, and neuroprotection, due to their versatility in modulating different biological pathways.^[^ ^167^ ^]^ In GBM treatment, their capacity to modulate the TME, inhibit tumor growth, and enhance the efficacy of existing therapies holds tremendous promise. By focusing on nanoenzymes in this review, we aim to highlight their unique advantages and the latest advancements in their development for GBM therapy, thereby providing insights into how they can address the current challenges in GBM treatment.

### 4.1 Nanoenzymes‐Enhanced Radiation Therapy

RT is a mainstay of GBM treatment, often administered post‐surgery to target residual tumor cells. However, the high doses of ionizing radiation required to achieve therapeutic effects in GBM can cause significant collateral damage to surrounding healthy brain tissue, leading to cognitive deficits and radiation‐induced brain injury (RIBI). The need for therapeutic strategies that can not only enhance the efficacy of radiation therapy but also protect healthy tissue has driven researchers to explore the use of nanoenzymes as radiosensitizers and neuroprotective agents. Nanoenzymes possess unique physical and chemical properties that can sensitize tumor cells to radiation, improving treatment outcomes. Specifically, metallic nanomaterials with high atomic numbers can act as radiosensitizers by increasing the absorption of radiation. This leads to enhanced energy deposition in tumor cells, resulting in greater DNA damage and cell death. The electron leaps caused by radiation absorption release energy in the form of ROS, which further amplifies the cytotoxic effects. In this context, nanoenzymes offer a dual benefit: radio sensitization to boost the effectiveness of radiotherapy and enzyme‐like activities to modulate oxidative stress in favor of tumor cell destruction.

**Figure 3:**
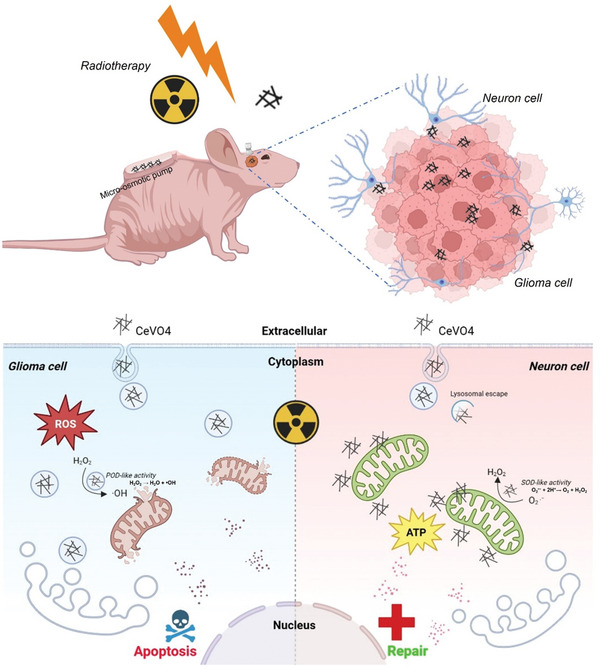
Catalytic‐therapeutic schematic of CeVO4. Under the action of ionizing radiation (IR), locally injected CeVO4 shows pH‐dependence for targeted killing of glioma cells and protection of neurons. Reproduced with permission.^[^ ^155^ ^]^ Copyright 2024, ACS.

For example, Han et al.^[^ ^155^ ^]^ developed cerium vanadate (CeVO4) nanoenzymes with multi‐enzyme activities to target GBM cells while providing neuroprotection against radiation‐induced damage. This nanoenzyme exhibited SOD‐like activity in neutral environments and peroxidase (POD)‐like activity in acidic conditions, which are characteristic of the TME. The selective enzyme activities of these nanoenzymes allow them to accumulate ROS within tumor cells, enhancing radiation‐induced cellular damage. In neuronal cells, the same nanoenzymes help mitigate mitochondrial damage induced by radiotherapy by reversing mitochondrial dysfunction and restoring the expression of anti‐apoptotic proteins such as BCL‐2. The application of CeVO4 nanoenzymes in combination with RT has shown remarkable results in preclinical models. In GBM‐bearing mice, the intertumoral administration of CeVO4 during RT led to significant tumor suppression and prolonged survival. Bioluminescence imaging revealed a marked reduction in tumor size in the group treated with CeVO4 and radiation, while Kaplan–Meier survival curves demonstrated that this combination extended the survival time of treated animals. Additionally, behavioral assessments of the animals indicated reduced brain edema and improved motor function, suggesting that CeVO4 not only enhances tumor cell killing but also alleviates radiation‐induced neurological damage. The use of nanoenzymes as radiosensitizers represents a promising strategy to overcome the limitations of traditional radiotherapy. Metallic nanoenzymes, such as those based on gold, silver, and cerium, have been extensively investigated for their radio‐sensitizing capabilities. Their high atomic numbers increase the likelihood of photon interaction during radiation, resulting in enhanced radiation absorption and greater energy deposition in tumor cells. This leads to the generation of more ROS, which induces DNA double‐strand breaks, the most lethal form of radiation‐induced damage. In addition to their radio‐sensitizing effects, nanoenzymes offer the advantage of multi‐enzyme activities that can be tailored to the specific needs of the TME. For example, nanoenzymes with CAT‐ or SOD‐like activities can modulate the oxidative stress within the tumor, creating conditions that are less favorable for tumor cell survival while protecting surrounding healthy tissues. This dual functionality makes nanoenzymes particularly attractive for GBM treatment, where the goal is to maximize tumor cell destruction while minimizing damage to the delicate brain tissue. (**Figure** **3**)

A significant challenge in GBM therapy is the protection of healthy brain tissue from the collateral effects of radiotherapy and chemotherapy. Radiation‐induced neuronal damage can lead to long‐term cognitive deficits, severely affecting the quality of life of survivors. Nanoenzymes, with their ability to modulate oxidative stress and promote neuronal protection, offer a unique solution to this problem. CeO_2_ nanoparticles, for example, have been extensively studied for their neuroprotective properties due to their reversible redox state between Ce^3+^ and Ce^4+^. This property allows CeO_2_ nanoparticles to act as ROS scavengers, reducing oxidative stress in healthy neuronal cells while promoting ROS accumulation in tumor cells. By protecting neurons from radiation‐induced apoptosis and mitochondrial dysfunction, nanoenzymes such as CeO_2_ have the potential to reduce the cognitive side effects associated with GBM treatment. Moreover, nanoenzymes can be engineered to interact with and modulate the TME, a critical factor in GBM progression. The GBM TME is characterized by hypoxia, immune suppression, and elevated levels of ROS, which promote tumor growth and resistance to therapy. Nanoenzymes with ROS‐regulating properties can disrupt this microenvironment by generating toxic levels of ROS within tumor cells while protecting normal cells from oxidative stress. This modulation of the TME not only enhances the therapeutic efficacy of radiation but also makes the tumor more susceptible to immune‐based therapies, offering the potential for synergistic treatment strategies.

Given their versatility, nanoenzymes have been investigated as components of combination therapies aimed at improving the overall efficacy of GBM treatment. For example, combining nanoenzymes with traditional chemotherapeutic agents or immune checkpoint inhibitors may provide a multifaceted approach to overcoming the inherent resistance of GBM to single‐modality therapies. Nanoenzymes can facilitate drug delivery, enhance radiosensitivity, and modulate the immune response within the TME. Preclinical studies have shown that nanoenzymes can enhance the delivery of TMZ, increasing its concentration within the tumor while reducing systemic toxicity. By acting as carriers or as part of nanoplatforms that respond to tumor‐specific stimuli, nanoenzymes enable more efficient drug release at the tumor site. Additionally, nanoenzymes that promote ROS accumulation within the TME can enhance the effectiveness of immunotherapy by promoting tumor antigen release and enhancing the infiltration of immune cells into the tumor.

### 4.2 Nanoenzymes‐Enhanced Photothermal Therapy

PTT has emerged as a minimally invasive treatment modality for cancer, utilizing photo‐absorbing agents to convert NIR light into localized heat, thereby inducing cancer cell apoptosis or necrosis. In the treatment of GBM, PTT offers the advantage of precise spatial control, minimizing damage to surrounding healthy brain tissue. However, the clinical application of PTT in GBM faces significant challenges. These include the limited penetration of NIR light through biological tissues, insufficient accumulation of photothermal agents at the tumor site due to the BBB, and the potential for heat shock protein expression by tumor cells, which can confer thermoresistance and reduce treatment efficacy. To address these limitations, the integration of nanoenzymes into PTT strategies has gained considerable attention. Nanoenzymes, with their intrinsic enzyme‐mimicking activities and nanoscale dimensions, can enhance PTT in several ways. They can improve the photothermal conversion efficiency, generating more heat upon NIR irradiation to effectively ablate tumor cells. Additionally, nanoenzymes can modulate the TME by alleviating hypoxia through catalytic decomposition of endogenous hydrogen peroxide into oxygen, thereby enhancing the sensitivity of tumor cells to PTT. The catalytic activities of nanoenzymes can also induce ROS generation, leading to synergistic photothermal and chemodynamical therapeutic effects. Furthermore, functionalization of nanoenzymes with targeting ligands facilitates BBB penetration and selective accumulation in GBM cells, overcoming one of the major obstacles in GBM therapy. By amplifying the therapeutic efficacy and addressing the inherent challenges of PTT in GBM, nanoenzyme‐enhanced photothermal therapy represents a promising avenue for improving patient outcomes and advancing toward clinical translation.

Nanoenzyme‐catalyzed CDT has emerged as a promising modality owing to its non‐invasiveness, potent cytotoxicity against tumor cells, and immunomodulatory effects. Synergizing CDT with PTT can further enhance therapeutic efficacy. TME is characterized by mild acidity, elevated levels of H₂O₂, and hypoxia—conditions that can induce tumor metastasis and diminish treatment effectiveness. To address these challenges, hydrogen peroxide‐responsive nanoenzymes have been investigated for tumor therapy. These nanoenzymes exhibit POD and OXD‐like activities, catalyzing the decomposition of H₂O₂ and oxygen within the TME to generate ROS. The photothermal effect can amplify the POD‐ and OXD‐like activities, leading to increased ROS production. Despite these advantages, current strategies combining CDT and PTT face several dilemmas in glioma treatment. First, most CDT approaches utilize metal ions (e.g., Fe^2^⁺ and Cu^2^⁺) as catalysts to enhance ROS generation. However, ROS are prone to rapid inactivation, and the catalytic activities of these metal ions are limited, especially under hypoxic and acidic conditions prevalent in the TME. Second, nanomaterials designed for combined CDT‐PTT therapy often consist of multiple functional units, complicating nanoparticle synthesis and drug formulation. Moreover, these nanomaterials typically exhibit poor water dispersibility and large particle sizes, which hinder their ability to cross the BBB for targeted glioma therapy. It has been reported that nanoparticles with smaller sizes and prolonged in vivo circulation can achieve enhanced BBB penetration and accumulate more effectively in brain tumors. Thirdly, glioma therapies like CDT and PTT predominantly rely on intravenous drug delivery, which is not well‐aligned with current clinical practices and may result in suboptimal efficacy and minimal immune activation. Given that surgical resection is the primary clinical intervention for GBM patients, postoperative intertumoral implantation of therapeutic agents offers a convenient strategy to complement existing treatments. Concentrated drug delivery at the tumor site can enhance therapeutic outcomes, reverse the immunosuppressive TME, and minimize systemic side effects. However, there is a scarcity of suitable intertumoral implants that are both highly malleable and capable of uniformly hosting CDT and PTT agents for postoperative treatment and prevention of glioma recurrence.

**Figure 4:**
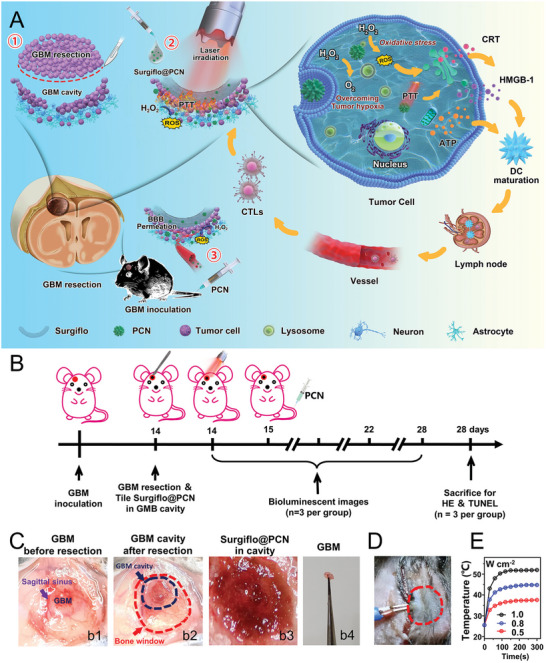
A) Schematic diagram of Surgiflo@PCN‐Mediated anticancer immunotherapy for inhibition of postoperative glioma recurrence in mice B) Schematic diagram of in vivo pharmacodynamic experimental design. C) Schematic diagram of nanosystems implanted into GBM. D,E) Temperature change curves of tumors irradiated by 808 nm laser at the same laser power density. Reproduced with permission.^[^ ^168^ ^]^ Copyright 2023, ACS.

To overcome these challenges, Nie et al.^[^ ^168^ ^]^ designed a hemostatic matrix system termed Surgiflo@PCN (porous palladium‐copper nanoclusters). This system comprises Surgiflo—a commonly used postoperative hemostatic material in neurosurgery with excellent biocompatibility and complete absorbability over time—and PCN nanoparticles that are uniform in size and highly dispersible in aqueous solutions. Surgiflo's malleability allows it to conform to the shape and size of the tumor cavity, ensuring comprehensive coverage. The alloy‐induced lattice distortion and hole‐mediated light‐trapping effects in PCN enhance their near‐infrared (NIR) photothermal properties for PTT and elevate their catalytic activities for ROS generation CDT and oxygen production to alleviate hypoxia. These nanoenzymes exhibit good stability and minimal metal‐ion dissolution within the TME. Under NIR irradiation, they can inhibit glioma growth without causing significant damage to normal tissues. Postoperatively, when the tumor cavity is filled with Surgiflo@PCN, the PCN nanoenzymes are gradually released. They display enzyme‐like activities—oxidase, peroxidase, and catalase facilitating ROS‐based oxidative stress and oxygen generation. This process mitigates the immunosuppressive state of the TME, induces immunogenic cell death (ICD), and activates circulating T cells, ultimately eradicating residual tumor cells and preventing recurrence. (**Figure** **4**)

**Figure 5:**
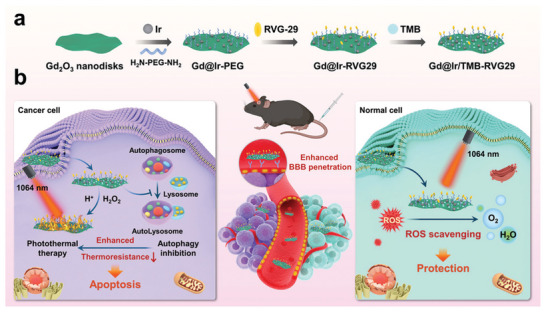
a) Schematic representation of the synthesis of Gd2O3@Ir/TMB‐RVG29 (G@IT‐R); b) G@IT‐R nanomachines interacting with cancer cells and normal cells for different PTT strategies. Reproduced with permission.^[^ ^157^ ^]^ Copyright 2023, Wiley‐VCH.

In another innovative study, Yin et al.^[^ ^157^ ^]^ focused on the design and development of a smart nanomachine for differential PTT of gliomas. While PTT is a promising modality for glioma treatment, its clinical application is limited by the potential for damaging adjacent healthy brain tissue and inducing inflammatory responses that may lead to tumor recurrence or metastasis. To overcome these limitations, the researchers developed a multifunctional nanomachine composed of Gd₂O₃@Ir/TMB‐RVG29 (G@IT‐R) hybrid nanomaterials. This system is engineered to deliver tumor‐specific PTT while simultaneously protecting normal brain tissue. The nanomachine is functionalized with the rabies virus glycoprotein‐29 (RVG29) peptide, which enables it to cross the BBB and selectively target glioma cells. Within the TME, iridium‐based nanoenzymes in the system act as logical control elements, triggering the amplified chromogenic reaction of 3,3′,5,5′‐tetramethylbenzidine. This reaction enhances the photothermal effect specifically in tumor cells. In contrast, in normal brain tissues, the nanomachine scavenges excess ROS generated by adverse therapies, thereby reducing inflammation and preventing collateral damage. The Gd₂O₃ component serves a dual purpose: it inhibits autophagy mechanisms in glioma cells, preventing them from repairing heat‐induced damage, and provides contrast enhancement for MRI, allowing real‐time monitoring of the treatment process. Key findings from this study indicate that the G@IT‐R nanomachine can effectively penetrate the BBB, selectively accumulate in glioma cells, and induce a potent photothermal effect. This system's ability to monitor treatment progression via MRI and inhibit tumor recurrence through anti‐inflammatory effects represents a significant advancement in nanomedicine design for brain cancer therapy. The research underscores the potential of smart, environmentally responsive nanotherapies to improve clinical outcomes for glioma patients by providing precise, targeted treatment while minimizing adverse effects on normal brain tissue. (**Figure** **5**)

In conclusion, the integration of nanoenzymes in photothermal therapy represents a promising avenue for GBM treatment. By addressing the limitations of current CDT and PTT strategies such as limited catalytic activity under hypoxic and acidic conditions, challenges in BBB penetration, and misalignment with clinical practices these advanced nanoenzyme‐based systems have the potential to improve therapeutic outcomes and prevent tumor recurrence.

### 4.3 Nanoenzymes‐Enhanced Immunotherapy

Immunotherapy has emerged as a transformative approach in cancer treatment, harnessing the body's immune system to recognize and eliminate tumor cells. In the context of GBM, however, immunotherapeutic strategies have faced significant challenges and have yet to achieve the remarkable successes seen in other malignancies such as melanoma and lung cancer. The highly immunosuppressive TME of GBM, characterized by the presence of regulatory T cells, myeloid‐derived suppressor cells, and tumor‐associated macrophages with an anti‐inflammatory phenotype, severely hampers the effectiveness of immune‐based therapies. Additionally, GBM exhibits profound heterogeneity and a lack of highly immunogenic neoantigens, which complicates the development of effective immune responses. The BBB further restricts the infiltration of immune cells and the delivery of immunotherapeutic agents to the tumor site. Despite these obstacles, the potential of immunotherapy in GBM remains substantial due to its ability to induce long‐lasting immune memory and systemic antitumor effects. Recent advancements have focused on overcoming the immunosuppressive TME, enhancing antigen presentation, and promoting the infiltration and activation of effector immune cells within the brain. Among these strategies, the integration of nanoenzymes into immunotherapeutic approaches presents a promising avenue to enhance efficacy. Nanoenzymes, with their unique catalytic activities and nanoscale properties, can modulate the TME by generating ROS, alleviating hypoxia, and promoting ICD, thereby converting the “cold” tumor milieu into an immunologically “hot” one. They can also facilitate the targeted delivery of immunomodulatory agents across the BBB and provide controlled release mechanisms to ensure sustained therapeutic concentrations at the tumor site. By addressing the critical barriers that have limited the success of immunotherapy in GBM, nanoenzyme‐enhanced immunotherapy holds the potential to elicit robust antitumor immune responses, improve patient outcomes, and pave the way for more effective combinational treatment strategies.

**Figure 6:**
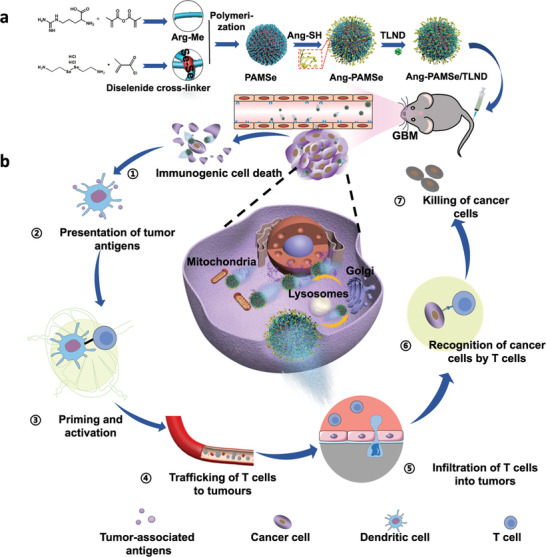
a) Preparation process of nanomotor. b) the schematic illustration of the cascading effect for enhanced immunotherapy of GBM. Reproduced with permission.^[^ ^169^ ^]^ Copyright 2023, Springer.

The GBM microenvironment is characterized by elevated levels of ROS and inducible nitric oxide synthase (iNOS). This unique biochemical milieu distinguishes tumor tissue from normal brain tissue, providing an opportunity to design targeting strategies that respond specifically to the GBM microenvironment. Leveraging this characteristic, Chen et al.^[^ ^169^ ^]^ designed and synthesized chemotactic nanomotors loaded with a brain endothelium‐targeting peptide (vasopressin‐2) and an antitumor drug, lonidamine modified with the mitochondria‐targeting moiety triphenylphosphine (TLND). In this innovative approach, the high concentrations of ROS and iNOS in the GBM microenvironment serve as chemotactic inducers, guiding the nanomotors toward the tumor site through chemotaxis. This strategy enables precise targeting across multiple biological barriers: from brain endothelial cells to tumor cells and ultimately to the mitochondria within those cells. The nanomotors are engineered to achieve a synergistic, multi‐step intervention in the tumor immune cycle through the release of nitric oxide (NO) and the action of the loaded drug TLND. During chemotaxis, the nanomotors generate high concentrations of NO, which functions as an inducer of ICD. This process enhances the production of tumor‐associated antigens, promoting the maturation of antigen‐presenting cells and the activation of T cells. NO also contributes to the normalization of aberrant vasculature within tumor tissues, improving the efficiency of T‐cell infiltration at the tumor site. By upregulating MMPs and reacting with ROS to produce peroxynitrite (ONOO⁻), NO facilitates the degradation of the tumor ECM, allowing deeper penetration of both immune cells and therapeutic agents. Furthermore, NO modulates macrophage polarization toward the pro‐inflammatory M1 phenotype, and inhibits the expression of programmed death‐ligand 1 (PD‐L1) on cancer cells, and Treg levels. These effects collectively enhance antitumor immunity. Simultaneously, TLND specifically inhibits aerobic glycolysis and disrupts energy metabolism in tumor cells by targeting their mitochondria. This disruption interferes with the metabolic symbiosis within the TME, helping to reverse immunosuppressive conditions commonly found in GBM. Experimental results confirmed that this therapeutic strategy not only effectively suppresses tumor growth but also establishes a robust immune memory effect, preventing tumor metastasis and recurrence. The synergistic action of NO and TLND represents a significant advancement in GBM immunotherapy, addressing both the challenges of drug delivery across the BBB and the activation of complex immune mechanisms within the TME. (**Figure** **6**)

**Figure 7:**
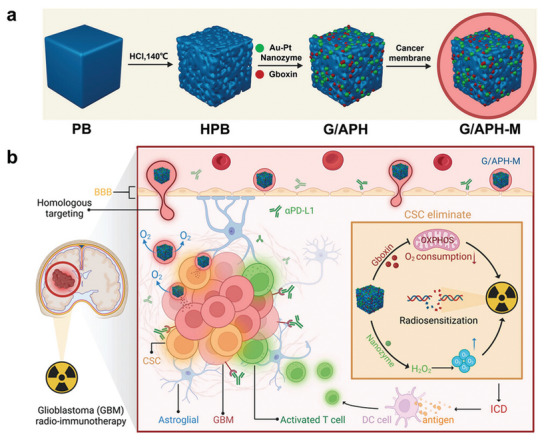
a) Schematic diagram of the preparation of G/APH‐M. b) Schematic of G/APH‐M‐based radiotherapy combined with anti‐PD‐L1 to enhance GBM radioimmunotherapy. Reproduced with permission.^[^ ^170^ ^]^ Copyright 2024, Wiley‐VCH.

In another innovative study, Xie et al.^[^ ^170^ ^]^ designed a biocompatible nanoplatform to enhance radioimmunotherapy for GBM. They utilized FDA‐approved hollow Prussian blue nanoparticles (PB NPs), known for their safety in treating thallium poisoning and radiation exposure, as drug delivery carriers. The positively charged Gboxin, a potent inhibitor of oxidative phosphorylation (OXPHOS) that targets mitochondrial respiration in GBM cells, was successfully loaded into these nanoparticles. By inhibiting OXPHOS and ATP generation, Gboxin mediates GBM cell‐specific death. To further enhance therapeutic efficacy, the authors co‐loaded gold‐platinum (Au‐Pt) bimetallic nanozymes into the system. These nanozymes exhibit catalase‐like activity, effectively decomposing H₂O₂ to generate oxygen, thereby alleviating tumor hypoxia—a common characteristic of the GBM microenvironment that contributes to radio resistance. The alleviation of hypoxia enhances the effectiveness of radiotherapy by increasing oxygen availability, which is crucial for the generation of radiation‐induced free radicals that damage tumor DNA. To facilitate effective penetration of the BBB, the Prussian blue nanoparticles were coated with cancer cell membranes derived from GBM cells. This biomimetic coating enables the nanoplatform to evade immune recognition and enhances its accumulation in tumor tissue via homotypic targeting mechanisms. The individual and synergistic effects of hypoxia alleviation and OXPHOS inhibition on the in vivo treatment of GBM were verified through bioluminescence imaging, body weight monitoring, and immunofluorescence analysis of tumor sections. The use of ICD induced by the combined Gboxin/Au‐Pt nanoplatform and radiotherapy elicited a robust antitumor immune response. Furthermore, the introduction of an immune checkpoint inhibitor, anti‐PD‐L1 antibody, amplified this immune response, as evidenced by an increase in CD8⁺ cytotoxic T lymphocytes and prolonged survival in treated animals. The biosafety of each treatment component was confirmed through blood chemistry and histological analyses, demonstrating minimal systemic toxicity and good biocompatibility. This study highlights the potential of combining nanotechnology with radioimmunotherapy to overcome the challenges associated with GBM treatment, such as tumor hypoxia, BBB penetration, and immune evasion. (**Figure** **7**)

While the activation of T cells and modulation of immune checkpoints represent emerging and promising strategies for GBM therapy, further research is needed to address challenges such as patient‐specific responses, the stability of nanoplatforms, and long‐term safety. Continued investigation into personalized treatment approaches, optimization of nanocarrier design for enhanced stability and targeting, and comprehensive evaluation of long‐term effects are essential steps toward advancing GBM immunotherapy toward clinical translation.

## 5 Nanoenzymes‐Enhanced Combination Therapy

The complex pathological features of GBM often render single‐modality treatments insufficient for effective management. Therefore, developing therapeutic strategies that integrate multiple methods and mechanisms is both an effective and rational approach to overcoming the limitations of conventional therapies.

**Figure 8:**
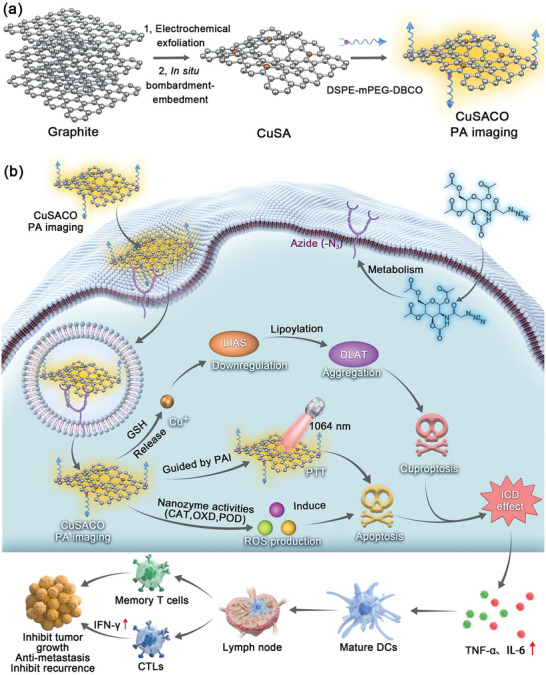
a) Schematic illustration of the general design of bioorthogonal single‐atom copper nanozymes (CuSACO). CuSA was prepared via an electrochemical exfoliation and in situ bombardment‐embedding strategy, followed by modification with DSPE‐mPEG2000‐DBCO to obtain CuSACO. b) CuSACO undergoes a bioorthogonal click reaction with azide groups on the tumor cell surface via a membrane‐flipping mechanism for efficient tumor targeting. The synergistic integration of nanozyme catalytic activity, cuproptosis, photothermal therapy, and immunotherapy effectively inhibits tumor growth, recurrence, and metastasis in vivo. Reproduced with permission.^[^ ^171^ ^]^ Copyright 2024, Wiley‐VCH.

Wu et al.^[^ ^171^ ^]^ ingeniously designed a novel bioorthogonal single‐atom copper nanozyme (CuSACO) aimed at synergistically combining nano catalytic therapy, PTT, cuproptosis, and immunotherapy for enhanced cancer treatment efficacy. Traditional cancer therapies such as surgery, chemotherapy, and radiotherapy often suffer from off‐target toxicity, recurrence, and metastasis. By leveraging the unique catalytic properties of single‐atom nanozymes (SAzymes), this study seeks to address these challenges and provide a highly targeted and efficient therapeutic strategy. The researchers synthesized CuSACO using an in situ exfoliation and embedding technique, allowing for the precise deposition of copper atoms onto graphene‐supported single‐atom structures. This synthesis method yielded a unique Cu–C₃ coordination structure that maximizes the exposure of active copper atoms, ensuring ≈100% utilization of these atoms. The structural design enables CuSACO to exhibit a variety of enzyme‐like activities, including CAT, OXD, and POD functions. These catalytic activities are triggered by the acidic TME and the presence of intracellular glutathione (GSH), leading to the generation of ROS that effectively destroy tumor cells through oxidative stress. A key innovation in the design of CuSACO is its bioorthogonal targeting capability. The nanozyme is modified with dibenzo cyclooctyne (DBCO), which selectively binds to azide groups on the surface of tumor cells through a click chemistry reaction. This mechanism allows CuSACO to specifically accumulate within tumors, catalyze ROS production, trigger photothermal effects under near‐infrared (NIR) irradiation, and induce cuproptosis—a copper‐dependent regulated cell death pathway. The release of copper ions into the TME further enhances therapeutic efficacy by downregulating lipoyl synthase (LIAS) and inducing proteotoxic stress through protein oligomerization, leading to efficient tumor cell death (**Figure** **8**). The synergistic integration of multiple treatment modalities in CuSACO highlights the potential of nanozymes in combination therapies. The catalytic activity of the nanozyme not only induces oxidative stress through ROS generation but also sensitizes tumor cells to PTT by disrupting cellular homeostasis. When exposed to NIR light at a wavelength of 1064 nm, CuSACO exhibits high photothermal conversion efficiency, resulting in precise PTT where the generated heat effectively kills tumor cells without damaging surrounding healthy tissue. The heat generated from PTT can further enhance the catalytic reactions by increasing the local temperature, thereby accelerating ROS production and amplifying oxidative damage to tumor cells. Moreover, the combination of catalytic therapy and PTT can promote ICD, which is crucial for activating antitumor immune responses. The release of tumor‐associated antigens and damage‐associated molecular patterns during ICD can stimulate dendritic cells and enhance antigen presentation, leading to the activation of cytotoxic T lymphocytes. This immune activation is further supported by the ability of CuSACO to induce cuproptosis, which involves copper‐induced cell death pathways that can modulate the immunosuppressive TME. By reprogramming the TME, nanozymes like CuSACO can enhance the efficacy of immunotherapy when used in combination. Additionally, nanozymes can synergize with radiotherapy by enhancing the generation of ROS within tumor cells. The elevated ROS levels can sensitize tumor cells to ionizing radiation, leading to increased DNA damage and apoptosis. Conversely, radiotherapy can disrupt the tumor vasculature and BBB, improving the penetration and accumulation of nanozymes in the tumor site. This bidirectional synergy amplifies the therapeutic outcomes of both modalities.

The combination of the catalytic and photothermal properties of CuSACO is central to its therapeutic efficacy. Upon exposure to NIR light at a wavelength of 1064 nm, the nanozyme exhibits high photothermal conversion efficiency, resulting in precise PTT where the generated heat effectively kills tumor cells without damaging surrounding healthy tissue. This photothermal effect not only enhances the direct killing of tumor cells but also works synergistically with catalytic therapy and cuproptosis to amplify the overall antitumor response. Moreover, the ability of CuSACO to induce ICD is a significant aspect of its therapeutic potential. The combined effects of nano catalytic therapy, ROS generation, PTT, and cuproptosis activate the immune system, leading to the release of pro‐inflammatory cytokines such as tumor necrosis factor‐alpha (TNF‐α), interleukin‐6 (IL‐6), and interferon‐gamma (IFN‐γ). This immune activation further enhances the eradication of tumor cells and helps prevent tumor metastasis and recurrence. The synergistic effects of nanozymes in combination therapies address the multifaceted challenges of GBM treatment. By integrating multiple therapeutic modalities, nanozymes can overcome tumor heterogeneity, reduce the likelihood of drug resistance, and target both the tumor cells and the supportive TME. For instance, the combination of PTT and immunotherapy not only directly destroys tumor cells through hyperthermia but also stimulates systemic immune responses that can target residual tumor cells and distant metastases. Similarly, combining radiotherapy with nanozyme‐enhanced catalytic therapy can amplify DNA damage in tumor cells while minimizing harm to healthy tissues. In preclinical models of breast cancer and glioma, CuSACO effectively inhibited tumor growth and significantly suppressed lung metastasis. Additionally, the imaging capabilities of CuSACO were emphasized, as the nanozyme can serve as a contrast agent for photoacoustic (PA) imaging, enabling real‐time, non‐invasive monitoring of its accumulation within tumors and the progress of treatment. In summary, the versatility of CuSACO positions it as an ideal candidate for next‐generation cancer therapies. By integrating catalytic activity, cuproptosis, PTT, and immunotherapy, CuSACO offers a highly targeted, efficient, and minimally invasive therapeutic approach that addresses many of the limitations of conventional cancer treatments. This study exemplifies how nanozymes can be engineered to synergize multiple therapeutic mechanisms, providing a comprehensive strategy for GBM treatment. Future research should focus on optimizing the design of nanozymes to maximize their synergistic effects in combination therapies. This includes exploring other therapeutic combinations, such as the integration of nanozymes with chemotherapy agents or gene therapy, and investigating the timing and sequencing of treatments to enhance efficacy. Understanding the interactions between nanozymes and the immune system, as well as their impact on the TME, will be crucial for developing effective combination therapies.

## 6 Challenges in Clinical Translation

Despite the significant promise that nanoenzymes hold for the treatment of GBM, their translation from the laboratory to clinical practice faces several critical challenges that must be meticulously addressed. One of the foremost limitations is the efficient targeting and delivery of nanoenzymes to the brain. The BBB, a highly selective barrier, restricts the passage of most therapeutic agents including nanoparticles thereby limiting drug accumulation at the tumor site. Overcoming this obstacle requires innovative strategies such as functionalizing nanoenzymes with ligands that facilitate receptor‐mediated transcytosis across the BBB, or utilizing techniques like focused ultrasound combined with microbubbles to temporarily disrupt the BBB and enhance nanoenzyme penetration. Achieving precise targeting of GBM cells while minimizing off‐target effects on healthy brain tissue is also crucial, as unintended distribution can lead to toxicity and reduced therapeutic efficacy. This necessitates the design of nanoenzymes with tumor‐specific recognition capabilities, such as decorating them with antibodies or peptides that bind to overexpressed receptors on GBM cells, and engineering stimuli‐responsive release mechanisms that activate in the unique TME.

Another critical limitation is the stability and quality control of nanoenzymes during production. Ensuring consistent manufacturing of nanoenzymes with uniform size, shape, surface properties, and catalytic activity is essential for predictable biological responses. The preparation of nanozymes often involves complex synthesis processes, such as chemical reduction, hydrothermal synthesis, or sol–gel methods, which can be challenging to control precisely. Variations in reaction conditions like temperature, pH, precursor concentrations, and reaction time can lead to inconsistencies in nanozyme properties. Achieving large‐scale production exacerbates these challenges, as scaling up reactions can alter the kinetics and thermodynamics of the processes involved. This requires the development of robust, reproducible synthesis protocols and comprehensive characterization techniques to monitor nanoparticle properties. Advanced techniques such as microfluidic reactors and continuous flow synthesis offer potential solutions by providing precise control over reaction parameters, enabling the production of nanozymes with consistent quality on a larger scale. Automation and in‐line monitoring systems can help maintain optimal conditions during synthesis, reducing batch‐to‐batch variability. Additionally, controlling the nucleation and growth processes through the use of surfactants or capping agents can aid in achieving uniform size and shape of nanozymes. Scaling up production from laboratory to industrial scale without compromising quality adds another layer of complexity. Industrial production must also address issues related to cost‐effectiveness, scalability, and compliance with regulatory standards. Collaborations with pharmaceutical manufacturers experienced in nanoparticle production can facilitate technology transfer and process optimization. Implementing Good Manufacturing Practice guidelines is crucial to ensure product quality and safety. Moreover, developing standardized protocols for nanozyme synthesis and functionalization will help in maintaining consistency across different production batches. Adopting scalable synthesis methods and collaborating with pharmaceutical manufacturers experienced in nanoparticle production can help address these challenges, but stringent quality control measures must be maintained throughout the process. Comprehensive characterization techniques, including transmission electron microscopy, dynamic light scattering, and zeta potential measurements, are essential for assessing nanozyme size distribution, morphology, and surface charge. Consistent catalytic activity must be verified through standardized assays to ensure therapeutic efficacy.

Safety and toxicity concerns present major hurdles in the clinical translation of nanoenzymes. The potential toxicity of nanozymes is influenced by their composition, size, shape, surface charge, and degradation products. Different nanozyme materials, such as metal oxides (e.g., Fe₃O₄, CeO₂) and metal nanoparticles (e.g., gold, silver, platinum), have varying biocompatibility profiles. Metal oxide nanozymes like Fe₃O₄ have been widely studied due to their peroxidase‐like activity and are generally considered biocompatible at low concentrations. However, at higher doses, they may induce oxidative stress, and inflammatory responses, or interfere with normal cellular functions. Metal nanoparticles such as gold and silver nanozymes offer excellent catalytic activities but raise concerns regarding long‐term toxicity and accumulation. Gold nanoparticles are often regarded as relatively inert and biocompatible; however, their nondegradable nature may lead to accumulation in organs like the liver and spleen over time. Silver nanoparticles possess antimicrobial properties but can induce cytotoxicity through the release of silver ions, leading to potential nephrotoxicity and hepatotoxicity. The metabolism and degradation pathways of nanozymes in the body significantly determine their long‐term safety. Nanozymes can be internalized by cells of the reticuloendothelial system, particularly in the liver and spleen, leading to accumulation and potential toxicity in these organs. Studies in animal models have shown that certain nanozymes can induce histopathological changes in the liver and kidneys, including inflammation, necrosis, and fibrosis, especially at high doses or with prolonged exposure. For example, in a study involving the administration of CeO₂ nanozymes in mice, significant accumulation was observed in the liver and spleen, with evidence of oxidative stress and inflammatory responses. Similarly, iron oxide nanozymes have been reported to cause alterations in liver enzyme levels and histological changes in renal tissues at high concentrations. These findings highlight the importance of thorough biocompatibility assessments and dose optimization. To mitigate these risks, designing nanozymes using biodegradable and biocompatible materials is essential. Employing materials that can be metabolized and excreted from the body reduces the likelihood of long‐term accumulation and toxicity. Surface modification of nanozymes with biocompatible polymers like polyethylene glycol can improve their circulation time while minimizing recognition and clearance by the immune system. Additionally, incorporating targeting ligands can enhance the selective uptake by tumor cells, reducing off‐target effects on healthy tissues. Conducting comprehensive in vitro and in vivo toxicological studies is crucial to evaluate the safety profiles of nanozymes. These studies should include assessments of acute and chronic toxicity, immunogenicity, genotoxicity, and carcinogenicity. Utilizing animal models that closely mimic human physiology allows for the evaluation of nanozyme biodistribution, metabolism, and excretion pathways. Monitoring biomarkers of organ function, such as liver enzymes and renal function indicators, provides insights into potential organ‐specific toxicities. Furthermore, long‐term studies are necessary to assess the potential for delayed adverse effects or cumulative toxicity. Regulatory agencies may require such data to ensure that nanozyme‐based therapies are safe for clinical use. Addressing these safety concerns through rigorous testing and optimization is paramount for the successful translation of nanozymes into clinical applications for GBM treatment.

Ethical considerations further complicate the clinical translation of nanoenzymes. Ensuring that patients fully understand the novel nature of nanoenzyme therapies, including potential risks and benefits, is imperative for informed consent. Clear communication without technical jargon and transparent discussions about uncertainties are necessary to facilitate patient understanding. Additionally, the high costs and limited availability of advanced nanoenzyme therapies may restrict access for some patients, potentially exacerbating health disparities. Developing cost‐effective manufacturing processes and advocating for healthcare policies that support equitable access are crucial to addressing these ethical concerns.

Regulatory and approval pathways represent another significant limitation. The lack of fully established regulatory frameworks for nanoenzyme‐based therapeutics leads to uncertainties in the approval process. Early engagement with regulatory agencies and adherence to existing guidelines for nanomaterials including characterization, manufacturing practices, and safety evaluations are necessary steps to navigate this complex landscape. Designing clinical trials that adequately assess safety and efficacy while addressing the unique properties of nanoenzymes poses additional challenges. Appropriate endpoint selection, careful patient selection considering GBM heterogeneity, and long‐term follow‐up are essential components of a robust clinical trial design.

Despite these limitations, the opportunities presented by nanoenzymes provide a compelling rationale for continued research and development in this area. Nanoenzymes offer enhanced efficacy through their catalytic activities, potentially improving drug potency and reducing required dosages. Their customizable nature enables precision medicine approaches, allowing targeting of specific molecular markers in GBM and aligning with personalized treatment strategies. Innovative delivery systems facilitated by nanoenzymes include controlled release and co‐delivery of multiple therapeutic agents, and their combination with other treatments like immunotherapy or radiotherapy can enhance overall therapeutic outcomes. Furthermore, their diagnostic capabilities allow for early detection and real‐time monitoring of treatment efficacy, which may lead to more timely interventions and better patient management. Improved efficacy and reduced side effects could also result in decreased overall healthcare costs by minimizing hospital stays and additional treatments. Balancing these challenges with the potential benefits is critical to the successful translation of nanoenzyme‐based therapies from the laboratory to the clinic. Systematic efforts to address limitations related to brain targeting, manufacturing consistency, safety, ethical considerations, and regulatory compliance are essential. Collaborative endeavors among scientists, clinicians, industry partners, regulators, and ethicists will be pivotal in overcoming these obstacles. By meticulously addressing these challenges, nanoenzymes hold the potential to revolutionize GBM treatment, offering hope for significantly improved patient outcomes and paving the way for their application in other challenging cancers.

## 7 Conclusion and Perspectives

Nanoenzymes represent a promising frontier in the treatment of GBM, offering unique enzyme‐mimicking activities that can effectively complement existing therapeutic modalities such as PTT and radiotherapy. By leveraging their catalytic properties, nanoenzymes can enhance ROS generation, leading to improved cancer cell destruction. When combined with precise brain‐targeting ligands, these nanoenzymes achieve enhanced efficacy while minimizing off‐target effects, thereby addressing one of the critical challenges in GBM therapy.

Among the various classes of nanoenzymes, peroxidase‐mimicking nanoenzymes have shown significant effectiveness in GBM treatment. These nanoenzymes, often based on materials like iron oxide nanoparticles, catalyze the conversion of endogenous hydrogen peroxide into highly reactive hydroxyl radicals. This process induces oxidative stress selectively in tumor cells, leading to apoptosis and necrosis. Catalase‐mimicking nanoenzymes are also effective, particularly in alleviating hypoxia within the TME by decomposing hydrogen peroxide into water and oxygen. This oxygen generation enhances the efficacy of oxygen‐dependent therapies such as radiotherapy and PTT. Additionally, SOD‐mimicking nanoenzymes can mitigate oxidative damage to healthy tissues by converting superoxide radicals into less harmful molecules, thereby reducing side effects associated with conventional treatments. The application of nanozymes in neurosurgical procedures offers unique advantages in minimally invasive GBM treatment. Nanozymes can be integrated into neurosurgical interventions to improve surgical precision and reduce trauma. For instance, nanozymes conjugated with imaging agents can provide real‐time intraoperative visualization of tumor margins, aiding surgeons in distinguishing between malignant and healthy tissue. This enhances the accuracy of tumor resection while preserving critical brain structures, potentially reducing neurological deficits post‐surgery. Furthermore, nanozymes can be delivered directly to the tumor site during surgical procedures, enabling localized catalytic therapy that targets residual tumor cells. This intraoperative administration can help reduce recurrence rates by eliminating microscopic diseases often left behind due to the infiltrative nature of GBM. Preclinical studies have demonstrated that nanozymes administered in conjunction with surgery can significantly inhibit tumor regrowth and prolong survival in animal models. Nanozymes also facilitate minimally invasive therapeutic approaches such as magnetic hyperthermia. Iron oxide nanozymes can generate localized heat when subjected to an external magnetic field, inducing tumor cell death while sparing surrounding healthy tissue. This technique can be applied via stereotactic injection, minimizing surgical trauma and offering a therapeutic option for patients who are not candidates for extensive surgery. In addition, nanozymes can modulate the postoperative TME to support tissue regeneration and repair. By reducing oxidative stress and inflammation through their catalytic activities, nanozymes can promote healing and reduce complications associated with neurosurgical interventions.

Innovative delivery systems, such as lipid‐based nanoparticles and other nanocarriers, have been developed to improve brain penetration and enable controlled drug release. These systems facilitate the traversal of the BBB, enhancing the delivery of nanoenzymes to the tumor site and resulting in more effective treatments. Moreover, nanoenzymes can be integrated with other therapeutic modalities, including immunotherapy and chemotherapy, to create synergistic effects that amplify antitumor responses. Their inherent diagnostic capabilities also offer potential for early detection and intervention, contributing to personalized medicine approaches. Combining nanoenzymes with other emerging technologies, such as CRISPR‐Cas9 gene editing and advanced imaging techniques, holds significant potential to further enhance therapeutic outcomes.^[^ ^172^ ^]^ The integration of nanoenzymes with CRISPR technology can facilitate targeted gene editing within GBM cells. Nanoenzymes can be engineered to deliver CRISPR components across the BBB efficiently, enabling precise modification or silencing of oncogenes and tumor suppressor genes involved in GBM progression.^[^ ^173^ ^]^ This dual‐action approach allows for simultaneous disruption of tumor growth pathways and induction of cytotoxic effects, potentially overcoming resistance mechanisms and improving treatment efficacy. Advanced imaging techniques, such as MRI and positron emission tomography (PET), can be enhanced through the use of nanoenzymes. Due to their unique physical and chemical properties, nanoenzymes can serve as contrast agents, improving the visualization of tumor boundaries and facilitating real‐time monitoring of therapeutic responses. The incorporation of imaging functionalities into nanoenzyme platforms supports the development of theragnostic agents, which combine therapeutic and diagnostic capabilities in a single system. This integration enables clinicians to personalize treatment plans and adjust therapies based on the patient's specific tumor characteristics and progression. However, despite these advancements, nanoenzymes face significant challenges that must be addressed before clinical translation can be realized. Achieving consistent and high‐quality production of nanoenzymes is paramount, as variations in synthesis can lead to discrepancies in therapeutic efficacy and safety. Ensuring the stability of nanoenzymes and maintaining their biological activity under physiological conditions are also critical concerns. The BBB remains a formidable obstacle, as it limits the delivery of therapeutic agents to the central nervous system. While strategies to enhance BBB penetration are being explored, there is a need for more effective and clinically viable solutions.

Ethical and safety considerations are essential and require thorough assessment. Potential long‐term toxicity, immunogenicity, and biocompatibility of nanoenzymes must be rigorously evaluated through comprehensive preclinical and clinical studies. Regulatory approval processes necessitate the development of standardized protocols for the clinical use of nanoenzymes, including clear guidelines on manufacturing practices, quality control, and safety evaluations. Addressing these regulatory hurdles is critical for the successful integration of nanoenzyme‐based therapies into clinical practice. In the broader context, standard treatments for glioma remain limited due to several key factors. This invasiveness leads to high recurrence rates even after aggressive surgical interventions. BBB is a specialized structure that regulates the transport of molecules into the brain parenchyma, hindering the delivery of most chemotherapeutic agents used in treating primary brain tumors. This limitation not only reduces the therapeutic effect but also increases the risk of systemic toxicity and side effects. Third, glioma cells possess robust DNA repair mechanisms that diminish the efficacy of chemotherapeutic agents and ionizing radiation. Additionally, glioma cells can develop various forms of intrinsic resistance to different therapies such as DNA damage checkpoint activation, low proliferation rates, and resistance within tumor stem cell subpopulations counteracting the cytotoxic effects of treatment. These factors collectively present significant challenges to achieving a complete cure for glioma with current standard therapies. Conventional drug delivery systems have inherent limitations, including limited targeting capabilities, low therapeutic indices, poor aqueous solubility, and the induction of drug resistance. Nanoparticle‐based drug delivery systems have been designed to overcome these drawbacks, serving as effective drug carriers with distinct advantages. These include the solubilization of hydrophobic drugs, extended circulation times in the bloodstream, and enhanced targeting of specific tissues or tumor sites. Nanoparticle‐based systems such as dendrimers, liposomes, self‐assembled peptides, water‐soluble polymers, and block copolymer micelles have rapidly evolved and are employed across various areas of biomedicine. Some of these nanocarriers are already in clinical use, demonstrating their potential for therapeutic applications.

Immunotherapeutic approaches have been actively investigated for the treatment of invasive gliomas. However, clinical studies have yielded disappointing results thus far. Sustained responses from major trials involving immunotherapies such as immune checkpoint inhibitors (ICIs), vaccines, chimeric antigen receptor T‐cell (CAR‐T) therapies, and oncolytic viruses remain rare. The primary reasons for these outcomes are multifactorial and include the highly immunosuppressive TME, a lack of specific and immunogenic tumor antigens, heterogeneity and plasticity at the single‐cell level, and limited drug penetration across the BBB. To overcome these obstacles, the development of nanomedicines capable of penetrating the BBB and improving immune cell transport and sensitivity to immunomodulatory therapies is imperative. Combining strategies involving immunotherapy, molecularly targeted therapies, and radiotherapy may enhance therapeutic efficacy. Additionally, in situ administration of immunotherapeutic agents within the surgical cavity post‐glioma resection can bypass BBB obstruction and activate robust immune responses. Finally, it is important to acknowledge that a significant proportion of nanoparticles accumulate and are sequestered within the reticuloendothelial system (RES) following systemic administration. This phenomenon leads to decreased delivery of nanoparticles to target diseased tissues and may result in non‐selective accumulation that poses toxicity risks to normal cells. Therefore, successful clinical translation of nanomedicine requires a comprehensive understanding of the in vivo transport, metabolism, and clearance of nanoparticles. Strategies to regulate these processes are essential to achieve highly effective therapeutic outcomes while mitigating associated adverse effects.

**Figure 9:**
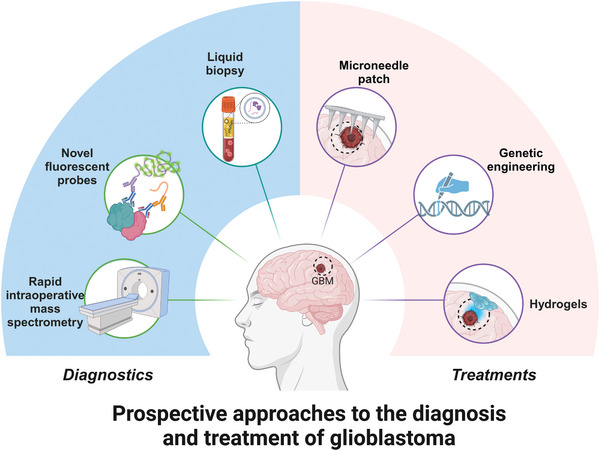
Schematic representation of potential future approaches to the diagnosis and treatment of GBM.

Nanoenzymes represent a transformative opportunity in GBM treatment, overcoming the shortcomings of existing therapies and introducing innovative mechanisms of action. By mimicking enzymatic activities, nanoenzymes can modulate TME and selectively induce cytotoxicity in cancer cells. Integrating these agents with advanced drug delivery platforms greatly enhances their therapeutic index, enabling precise targeting of the tumor while minimizing systemic toxicity. However, realizing the full clinical potential of nanoenzymes in GBM therapy demands focused research in several key areas. First, optimizing nanoenzyme design to improve BBB penetration without compromising stability and efficacy remains a central challenge. Solutions may include developing multifunctional nanocarriers capable of navigating the brain's complex environment and releasing therapeutic agents in a controlled manner. When combined with CRISPR‐Cas9 gene editing technology, nanoenzymes can deliver these editing tools across the BBB to directly modify oncogenic pathways or restore tumor suppressor gene function within cancer cells. Such a dual‐action approach addresses both genetic and microenvironmental factors underlying GBM progression. Second, integrating nanoenzymes with advanced imaging modalities offers new avenues for enhancing diagnostic accuracy and treatment monitoring. By engineering nanoenzymes to serve as contrast agents for MRI, PET, or fluorescence imaging, clinicians can visualize nanoenzyme distribution, assess BBB penetration, and track therapeutic responses in real time, thereby enabling personalized, adaptive treatment strategies. Third, artificial intelligence and machine learning can significantly accelerate nanoenzyme development. By modeling nanoenzyme behavior in biological systems, optimizing design parameters, and personalizing treatments based on patient‐specific factors, AI can identify the most effective combinations of nanoenzymes and other emerging technologies. Comprehensive preclinical studies are essential to ascertain long‐term safety profiles, pharmacokinetics, biodistribution, and potential immunogenicity, while close collaboration among researchers, clinicians, and regulatory bodies will help establish standardized manufacturing, quality control, and clinical evaluation protocols. Looking ahead, a new generation of diagnostic and therapeutic innovations (**Figure** **9**) is set to reshape GBM management by enhancing precision, personalization, and patient outcomes. Rapid intraoperative mass spectrometry provides real‐time molecular characterization of surgical margins, guiding more accurate tumor resection and minimizing damage to healthy tissue. Novel fluorescent probes help distinguish malignant from benign tissue intraoperatively, improving surgical outcomes and reducing residual disease. Beyond the operating room, non‐invasive liquid biopsies enable continuous monitoring of tumor dynamics, allowing for timely adjustments to therapy as the disease evolves. On the therapeutic side, microneedle‐based delivery systems present a minimally invasive strategy to bypass the BBB, ensuring highly localized drug delivery and reduced systemic toxicity. CRISPR‐Cas9 gene editing tools offer the potential to correct genetic aberrations at their source, paving the way for truly personalized interventions. Biocompatible hydrogels, placed intracranially after surgery, can continuously release therapeutics into the tumor cavity, sustaining effective drug concentrations over time without repeated administrations. Collectively, these emerging technologies ranging from intraoperative diagnostics to gene editing and novel delivery platforms can work synergistically to transform GBM treatment. By integrating nanoenzymes into this evolving paradigm, clinicians can refine surgical precision, monitor disease progression non‐invasively, and deploy highly targeted therapies that respond dynamically to tumor changes. Ultimately, combining these breakthroughs may lead to significantly prolonged survival, improved quality of life, and steady progress toward long‐term disease control in GBM patients.

In conclusion, while considerable obstacles remain, the advancements in nanoenzyme research provide a compelling vision for the future of GBM therapy. By harnessing the unique properties of nanoenzymes and integrating multidisciplinary approaches, it is possible to develop highly effective, targeted treatments that improve patient outcomes. Continued investment in research, collaboration across scientific domains, and a commitment to addressing clinical translation challenges will be pivotal in transforming the potential of nanoenzymes into tangible therapeutic realities for GBM patients.

## Conflict of Interest

The authors declare no conflict of interest.
